# Highly Porous Au–Pt Bimetallic Urchin-Like Nanocrystals for Efficient Electrochemical Methanol Oxidation

**DOI:** 10.3390/nano11010112

**Published:** 2021-01-06

**Authors:** Heon Chul Kim, Jong Wook Hong

**Affiliations:** 1 Department of Chemistry, University of Ulsan, Ulsan 44776, Korea; gjscjf9426@ulsan.ac.kr; 2 Energy Harvest-Storage Research Center (EHSRC), University of Ulsan, Ulsan 44776, Korea

**Keywords:** bimetallic nanocrystals, porous nanostructures, electrocatalysis, methanol oxidation reaction

## Abstract

Highly porous Au–Pt urchin-like bimetallic nanocrystals have been prepared by a one-pot wet-chemical synthesis method. The porosity of urchin-like bimetallic nanocrystals was controlled by amounts of hydrazine used as reductant. The prepared highly porous Au-Pt urchin-like nanocrystals were superior catalysts of electrochemical methanol oxidation due to high porosity and surface active sites by their unique morphology. This approach will pave the way for the design of bimetallic porous materials with unprecedented functions.

## 1. Introduction

The Noble metals are active and stable materials that can be applied to various electrocatalysis [1,2,3,4,5]. Recently, noble metal-based nanocrystals (NCs) have been widely studied owing to their unique physical and chemical properties, which are different from those of their parent metals in bulk [6,7,8,9]. In particular, Pt-based NCs have been extensively investigated as a potential candidate material for various electrochemical reactions including electrochemical fuel oxidation, oxygen reduction, sensing, and hydrogen evolution by water splitting [10,11,12,13,14]. Meanwhile, given Au is catalytically more inert than other metals, bimetallic materials prepared by integrating Au into active metals exhibit excellent catalytic performances in various electrochemical reactions [15,16]. In this context, recently developed Au–Pt bimetallic NCs show distinct catalytic effects compared with single Pt and Au NCs, which has aroused fundamental interest for enhanced functionalities and catalysis applications [17,18,19]. In particular, Au–Pt bimetallic NCs exhibit enhanced electrocatalytic performance in fuel oxidation reactions in polymer electrolyte membrane fuel cells (PEMFC) because surface Au atoms can suppress the formation of carbonaceous molecules, which can be strongly adsorbed on the surface of NCs [16,20,21,22]. To fully exploit the advantages of bimetallic NCs for electrocatalysis, highly porous structures are promising candidates due to their high-surface area-to-volume ratios [23,24,25,26,27]. Therefore, the development of a facile strategy for the synthesis of highly porous Au–Pt bimetallic NCs is significant for achieving enhanced electrocatalytic performance. Nevertheless, the strategy for preparing Au–Pt bimetallic NCs with controlled shapes is still challenging.

Herein, we report a simple strategy for the synthesis of highly porous, urchin-like bimetallic Au–Pt NCs (Au–Pt HP-UNCs) by manipulating the reduction kinetics of Au and Pt precursors by controlling the amount of hydrazine used as reductant. Hydrazine has shown stronger reducing capability than convectional reductants such as ascorbic acid and citric acid, which are widely used for the synthesis of noble metal NCs. Due to the high reducing power of hydrazine, both Au and Pt precursors can be expected to grow rapidly, thereby enabling the formation of bimetallic Au–Pt HP-UNCs. Metallic, porous, urchin-like nanostructures can provide not only large surface area but also numerous surface active sites, which are significant for electrochemical applications. Unique properties of the prepared bimetallic Au–Pt HP-UNCs provide them with enhanced activity toward methanol oxidation reaction (MOR). Additionally, incorporating Au in Pt materials increases the electrochemical stability compared with commercial Pt/C, which reveals the promising potential of the bimetallic Au–Pt HP-UNCs as catalysts in direct methanol fuel cell systems.

## 2. Materials and Methods

### 2.1. Chemicals

Tetrachloroauric(III) acid trihydrate (HAuCl_4_, 99.9%), potassium tetrachloroplatinate(II) (K_2_PtCl_4_, 99.0%), cetyltrimethylammonium chloride solution (CTAC, solution in water, 25 wt%), hydrazine monohydrate (64.0–65.0%), KOH (≥85%), methanol (≥99.8%), and Nafion resin solution (5 wt%) were purchased from Sigma-Aldrich (St. Louis, MO, USA). Pt/C (40 wt%, average Pt particle size = 3 nm) was purchased from Alfa Aesar (Haverhill, MA, USA). Deionized water (18.2 MΩ cm) was employed to prepare the reaction solutions.

### 2.2. Synthesis of Au–Pt HP-UNCs

In a typical synthesis of Au–Pt HP-UNCs, 1.0 mL of HAuCl_4_ (5 mM) and 1.0 mL of K_2_PtCl_4_ (5 mM) were added into 5 mL of CTAC (50 mM), and then 50 μL of hydrazine (200 mM) was added into the mixture and sonicated for ~1 min. The resulting mixture was kept at 100 °C for 1 h in a conventional oven. Subsequently, the products were collected by centrifugation and washed two times with ethanol.

### 2.3. Synthesis of Rounded Au–Pt UNCs

In a typical synthesis of rounded Au–Pt UNCs, 1.0 mL of HAuCl4 (5 mM) and 1.0 mL of K_2_PtCl_4_ (5 mM) were added into 5 mL of CTAC (50 mM), followed by addition of 2.0 mL of hydrazine (200 mM). The resulting mixture was sonicated for ~1 min and then kept at 100 °C for 1 h in a conventional oven. Subsequently, the products were collected by centrifugation and washed two times with ethanol.

### 2.4. Electrocatalysis

Electrochemical measurements were conducted in a three-electrode cell using a Bio-Logic EC-Lab SP-300 (Bio-Logic SAS, Claix, France). Pt wire and Hg/HgO (1 M NaOH) were used as the counter and reference electrodes (ALS Co., Tokyo, Japan), respectively. All electrochemical data were obtained at room temperature. To prepare the working electrode, 10 mL of catalyst ink containing 1 mg of Pt according to inductively coupled plasma-optical emission spectrometry (ICP-OES) was dropped onto a glassy carbon electrode (GCE, diameter: 5 mm) and then dried at room temperature. The dried GCE was cleaned electrochemically by 50 potential cycles between −0.857 and 0.393 V vs. Hg/HgO at a scan rate of 50 mV s^−1^ in 0.1 M KOH. Electrolyte solutions were purged with N_2_ gas for 30 min before performing electrochemical experiments. CVs of all the catalysts were obtained between −0.857 and 0.393 V vs. Ag/AgCl at a scan rate of 50 mV s^−1^ in 0.1 M KOH or 0.1 M KOH/0.5 M methanol.

### 2.5. Characterization

Transmission electron microscopy (TEM) and scanning electron microscopy (SEM) images of the prepared catalysts were obtained using JEOL JEM-2100F, and JEOL JEM-7610F microscopes (JEOL Ltd., Tokyo, Japan), respectively. ICP-OES measurements were performed using a Spectroblue-ICP-OES (AMETEK Inc., Berwyn, PA, USA). X-ray diffraction (XRD) measurements were conducted on a Rigaku D/MAX2500 V/PC diffractometer (Rigaku, Tokyo, Japan).

## 3. Results and Discussions

**Figure 1:**
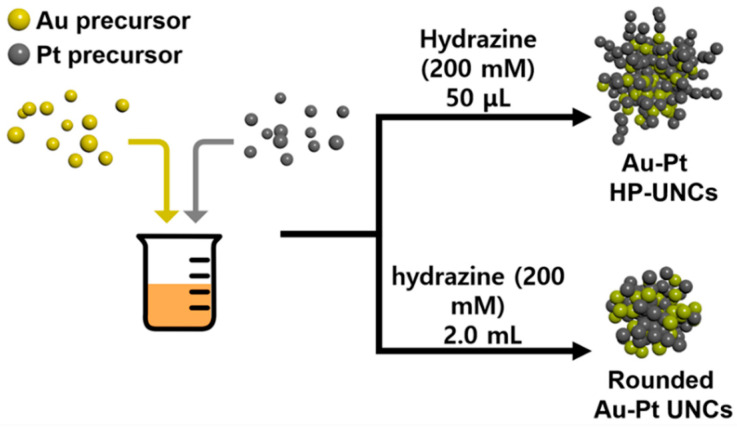
Schematic illustration for Au–Pt HP-UNCs and rounded Au–Pt UNCs.

The synthetic procedures for Au–Pt HP-UNCs are shown in Figure 1. Au–Pt HP-UNCs were produced by reducing Au and Pt precursors in a reaction mixture including hydrazine and CTAC as reductant, and surfactant, respectively.

**Figure 2:**
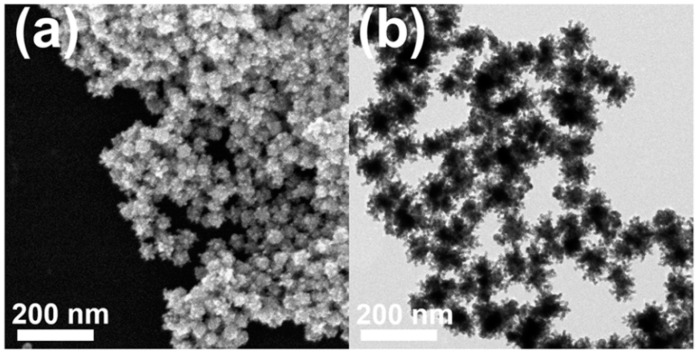
(**a**) SEM and (**b**) TEM images of Au–Pt HP-UNCs.

**Figure 3:**
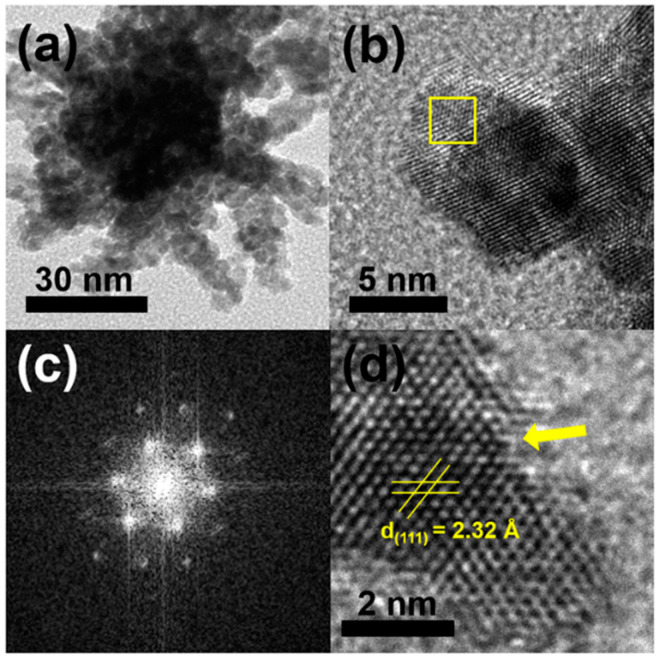
TEM images of; (**a**) an Au–Pt HP-UNC; and (**b**) branch of an Au–Pt HP-UNC. (**c**) FFT pattern obtained from yellow square in (**b**). (**d**) HR-TEM image in the branch of an Au–Pt HP-UNC.

Typically, a HAuCl_4_/K_2_PtCl_4_ mixture in a molar ratio of 1:1, CTAC, and hydrazine were added into deionized water, and the reaction mixture was heated at 100 °C for 1 h. Figure 2a,b display typical scanning electron microscopy (SEM), and transmission electron microscopy (TEM) images of the products, respectively, in which highly porous UNCs with average branch thickness of 6.2 ± 0.9 nm and average diameter size of 37.4 ± 5.5 nm can be observed as major products. Figure 3a,b show the high porosity of the UNCs from considerably porous dendritic branches and the fast Fourier transform (FFT) pattern obtained from a square region of a highly porous UNC reveals that the synthesized nanostructures are highly crystalline (Figure 3c). In addition, a d-spacing of 2.32 Å is observed between adjacent lattice fringes, which corresponds to the (111) planes of face centered cubic (fcc) Au–Pt (Figure 3d) [25] and demonstrates the Au–Pt bimetallic nature of the HP-UNCs. Noticeably, the high-resolution TEM (HRTEM) image displayed in Figure 3d shows many low-coordinated surface atoms at the branches of the Au–Pt HP-UNCs, which can serve as highly efficient active sites for electrochemical fuel oxidation reaction [26].

**Figure 4:**
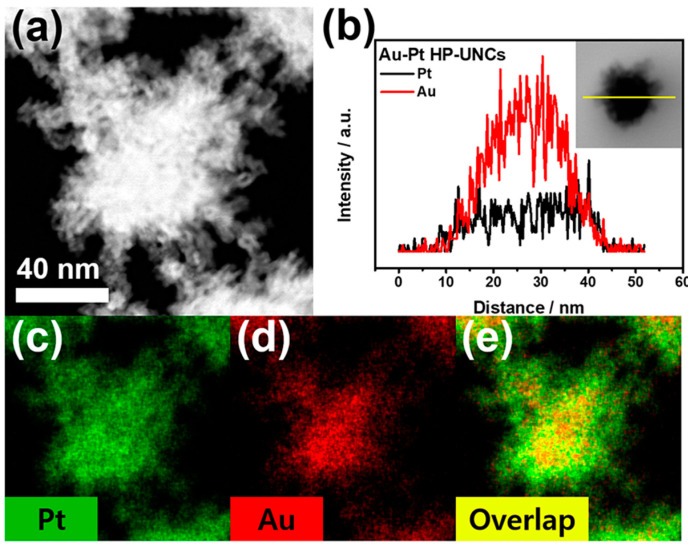
(**a**) HAADF-STEM, (**b**) cross-sectional compositional line profiles, and (**c**–**e**) HAADF-STEM-EDS elemental mapping images of an Au–Pt HP-UNC.

**Figure 5:**
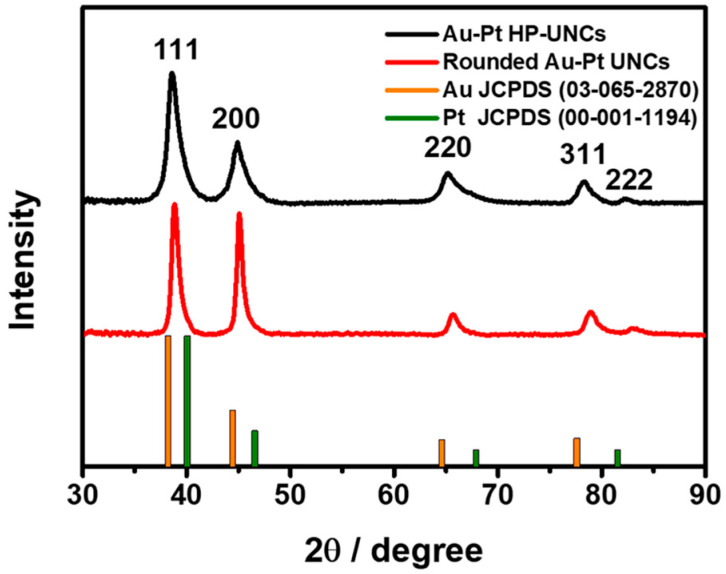
X-Ray Diffraction (XRD) pattern of Au–Pt HP-UNCs and rounded Au–Pt UNCs. The positions of Au and Pt were taken from the JCPDS database.

To check the morphological and compositional structure of the products, high-angle annular dark-field scanning TEM (HAADF-STEM) and corresponding elemental mapping images (Figure 4a) and the compositional line profile on a single highly porous UNC (Figure 4b) were obtained by HAADF-STEM energy dispersive X-ray spectroscopy (HAADF-STEM-EDS). HAADF-STEM image of the Au–Pt HP-UNCs reveals the formation of branches with high porosity (Figure 4a). Meanwhile, the elemental mapping of Au and Pt and the compositional line profile on a single highly porous UNC demonstrate that the synthesized HP-UNCs is an Au–Pt alloy, in which Au is more abundant than Pt at the inner region of the NCs. Whereas, most surfaces of the HP-UNCs including branches are composed of Pt. The higher Au content in the inner part of the HP-UNCs is ascribable to the higher reduction potential of the Au precursor (AuCl_4_^−^, 1.002 V) compared with that of the Pt precursor (PtCl_4_^2−^, 0.755 V) [28]. Therefore, the nucleation of Au can be expected to occur initially by dominant reduction of Au ions and, then, HP-UNCs are formed by subsequent coreduction of residual Au and Pt precursors on the preformed Au seeds. Furthermore, the XRD pattern of the products exhibits diffraction peaks attributed to fcc Au and Pt references, which demonstrates the Au–Pt bimetallic nature (Figure 5). Based on Scherrer equation, the crystalline size of the HP-UNCs was determined to be 6.56 nm. The Au/Pt ratio of the entire HP-UNCs was determined to be 58:42 by ICP-OES.

**Figure 6:**
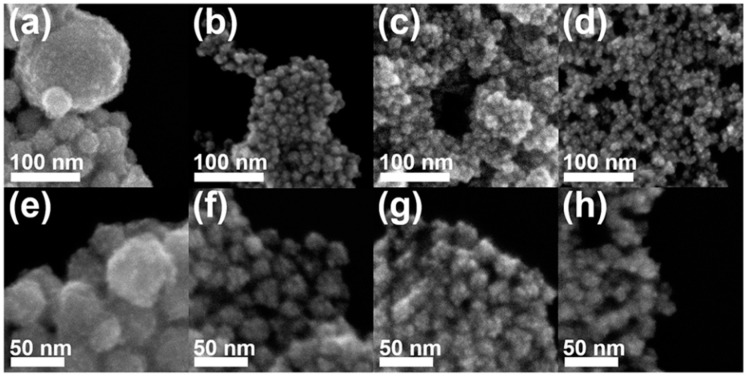
SEM images of Au–Pt NCs produced by (**a**,**e**) 10 μL, (**b**,**f**) 0.2 mL, (**c**,**g**) 2.0 mL, and (**d**,**h**) 5.0 mL of hydrazine (200 mM).

**Figure 7:**
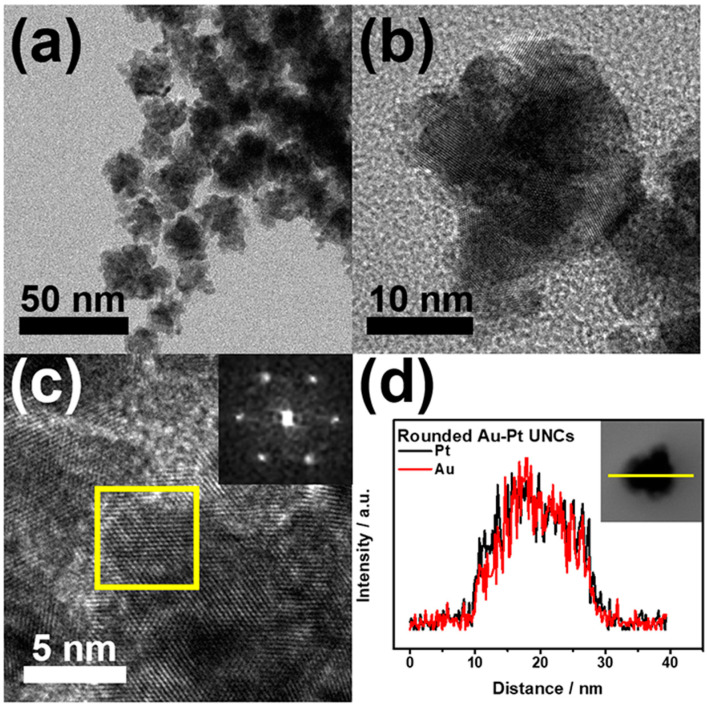
(**a**) TEM images of rounded Au–Pt UNCs. (**b**) TEM; and (**c**) high-magnification TEM images of a rounded Au–Pt UNC. The insets in (**c**) show an FFT pattern obtained from a rounded Au–Pt UNC. (**d**) Cross-sectional compositional line profiles of a rounded Au–Pt UNC.

Controlling the concentration of hydrazine is important for the formation of Au–Pt HP-UNCs. By evaluating the morphological change in Au–Pt NCs obtained in the presence of different amounts of hydrazine (Figure 6), we found that highly porous nanostructures were produced for a certain amount of hydrazine in the reaction mixture. The standard synthesis of the Au–Pt HP-UNCs was performed using 50 μL of hydrazine (200 mM). In contrast, large spherical NCs with an average size of 55.9 ± 9.5 nm were obtained when using 10 μL of hydrazine (200 mM), while keeping the other reaction conditions unchanged (Figure 6a,e). The formation of large spherical NCs can be attributed to a slow reduction rate of the metal precursor in the presence of a low concentration of hydrazine. Upon increasing the amount of hydrazine (200 mM) to 0.2, 2.0, and 5.0 mL, UNCs with rounded branches were formed (Figure 6b–d,f–h). However, their porosity was slightly lower than that of the standard Au–Pt HP-UNCs. To further investigate the morphological features of the porous products, the rounded UNCs produced in the presence of 2.0 mL of hydrazine (200 mM) were subjected to TEM analysis (Figure 7a,b). TEM images reveal that the porosity of the rounded UNCs is lower than that of standard Au–Pt HP-UNCs. The FFT pattern obtained from a square region of a rounded UNC shows high crystallinity of rounded UCNs (Figure 7c). Notably, an Au–Pt alloy nature can be observed in the entire NCs according to the compositional line profile on a single rounded UNC (Figure 7d). Taken together, these results suggest that hydrazine is an effective reductant for the synthesis of highly porous NCs and controlling the hydrazine concentration enables the tuning of the porosity and compositional structure of Au–Pt UNCs.

**Figure 8:**
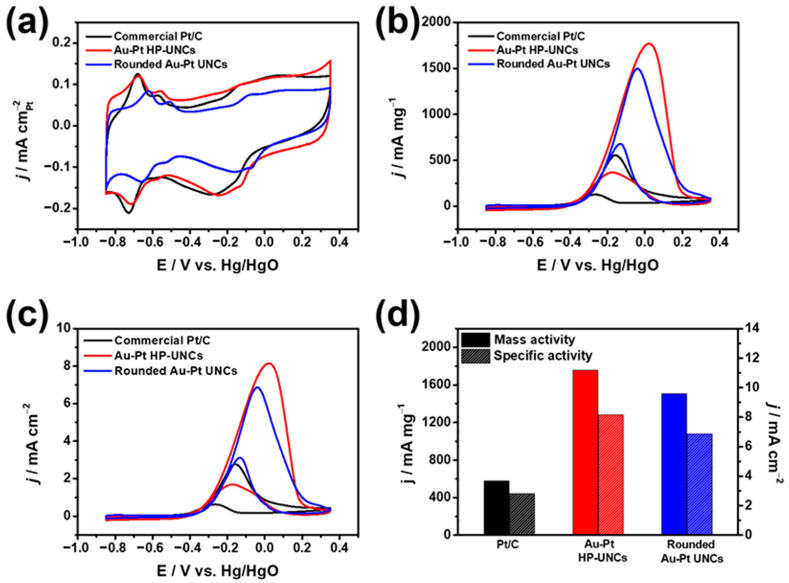
CVs obtained with various catalysts in (**a**) 0.1 M KOH and (**b**,**c**) 0.1 M KOH/0.5 M methanol at a scan rate of 50 mV s^−1^. Catalytic activities of different catalysts normalized by (**b**) mass of metal, and (**c**) ECSAs of catalysts. (**d**) Catalytic activities of various catalysts.

To investigate the influence of the porosity of the Au–Pt HP-UNCs on the electrocatalytic performance, MOR was selected as a model reaction. Electrocatalytic activities of the rounded Au–Pt UNCs and commercial Pt/C toward MOR were also measured to provide comparison. During the electrochemical measurements, the catalysts loaded on a GCE used as a working electrode were scanned between −0.857 and 0.393 V versus Hg/HgO in N_2_-saturated 0.1 M KOH or 0.1 M KOH/0.5 M methanol solutions. Figure 8a displays the CVs of the Au–Pt HP-UNCs, rounded Au–Pt UNCs, and commercial Pt/C catalysts. Electrochemically active surface areas (ECSAs) of the catalysts were determined using the hydrogen underpotential deposition (H_UPD_) analysis of the catalysts to be 21.7, 21.8, and 20.0 m^2^ g^−1^ for the Au–Pt HP-UNCs, rounded Au–Pt UNCs, and Pt/C catalysts, respectively. Although the size of the Au–Pt HP-UNCs is larger than that of the Pt/C catalysts (average diameter size: 5.4 ± 0.7 nm), their comparable ECSAs can be attributed to the high porosity of the dendritic branches. Figure 8b shows the CVs of the different catalysts obtained in 0.1 M KOH/0.5 M methanol. Peaks observed between −0.3 to 0.2 V vs. Hg/HgO can be attributed to oxidation of methanol on the surface of the catalysts. On the reverse scan, small peaks ascribable to the oxidation of adsorbed CO intermediate were observed at −0.4 to 0 V versus Hg/HgO for the Au–Pt HP-UNCs, rounded Au–Pt UNCs, and Pt/C catalysts. Apparently, the Au–Pt HP-UNCs show the largest MOR activity among the catalysts tested. Mass activities of the Au–Pt HP-UNCs, rounded Au–Pt UNCs, and Pt/C catalysts are 1759, 1509, and 579 mA mg^−1^, respectively (Figure 8b,c). Moreover, the current density of the Au–Pt HP-UNCs is the highest among the different catalysts. The specific current density normalized by ECSAs of the Au–Pt HP-UNCs was determined to be 8.17 mA cm^−2^, which is roughly 1.19 and 2.91 times higher than those of the rounded Au–Pt UNCs (6.87 mA cm^−2^), and Pt/C catalysts (2.81 mA cm^−2^), respectively (Figure 8d,e). The enhanced electrocatalytic activity of the Au–Pt HP-UNCs can be ascribed to their highly porous morphology, which provides larger active sites. Additionally, the presence of undercoordinated atoms in the Au–Pt HP-UNCs can effectively promote the electrochemical oxidation of methanol, thereby, enhancing the MOR activity compared with that of the rounded Au–Pt UNCs. Meanwhile, both types of Au–Pt UNCs exhibit better electrocatalytic activity than the Pt/C catalysts. This significant enhancement can be attributed to the presence of Au and Pt promoting the removal of carbonaceous poisoning intermediates such as CO from the surface of NCs. In addition, the forward/backward current (I_f_/I_b_) ratios of Au-Pt HP UNCs and Pt/C were compared to estimate the CO tolerance of the nanostructures. The Au-Pt HP-UNCs (4.78) showed higher I_f_/I_b_ ratio than Pt/C (4.11), which demonstrates the enhanced CO tolerance of Au-Pt HP-UNCs due to formation of Au-Pt bimetallic nanostructures. This high tolerance for such poisoning intermediates may be due to the ligand effect, which is associated with the modification of the electronic structures of Au and Pt by formation of Au–Pt bimetallic structures [29,30,31].

**Figure 9:**
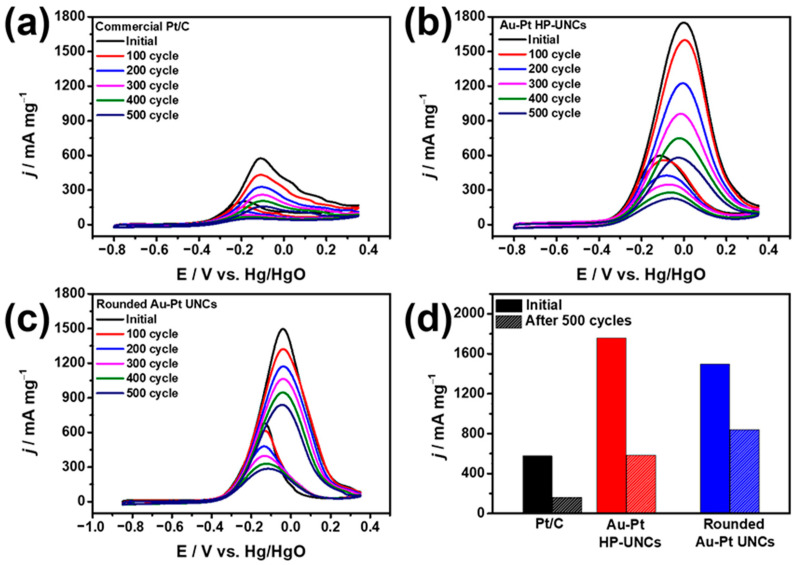
CVs obtained before and after MOR cycles for, (**a**) commercial Pt/C, (**b**) Au–Pt HP-UNCs, and (**c**) rounded Au–Pt UNCs in 0.1 M KOH/0.5 methanol at a scan rate of 50 mV s^−1^. Electrocatalytic stabilities of different catalysts in the (**d**) MOR.

The electrochemical stabilities of the Au–Pt HP-UNCs and rounded Au–Pt UNCs were estimated through accelerated durability test and compared with that of commercial Pt/C catalysts. Working electrodes, including the catalysts, were scanned between −0.85 and 0.3 V in N_2_-saturated 0.1 M KOH/0.5 M methanol. During the measurement, the decrease in mass activity of the catalysts was monitored for 500 cycle intervals. An attenuated decrease in the MOR activity of both Au–Pt HP-UNCs and rounded UNCs compared with that of Pt/C was observed (Figure 9). CVs of the catalysts obtained after 500 cycles show mass activities of 588, 850, and 349.7 mA mg^−1^ for Au–Pt HP-UNCs, rounded Au–Pt UNCs, and Pt/C catalysts, respectively, which indicate losses of 66.8%, 43.8%, and 72.5% in mass activity, respectively (Figure 9d). Enhanced electrocatalytic stabilities of the Au–Pt HP-UNCs and rounded Au–Pt UNCs compared to Pt/C can be attributed to the Au–Pt bimetallic structures. It is generally accepted that bimetallic catalysts including Au possess high tolerance to poisoning intermediates formed during electrochemical fuel oxidation [32,33]. Therefore, many bimetallic catalysts have shown enhanced catalytic stability compared with monometallic catalysts toward electrocatalytic reactions [34]. Considering this, it seems reasonable to assume that the higher stability of the Au–Pt UNCs stems from the presence of Au atoms, which reduce the adsorption of the poisoning intermediates. Interestingly, although the catalytic activity of the rounded Au–Pt UNCs was lower than that of the Au–Pt HP-UNCs, the former showed higher stability than the latter (Figure 9d). This might be due to the fact that the alloy surface of the rounded Au–Pt UNCs contains more Au atoms than the surface of the Au–Pt HP-UNCs, which is mainly composed of Pt. The high Au content in the surface of the Au–Pt UNCs could enhance the removal capability for poisoning intermediates by decreasing the adsorption energy of the intermediates. Therefore, the Pt surface atoms in the Au–Pt rounded UNCs can effectively function as MOR catalytic sites, thus, resulting in enhanced electrocatalytic stability of the Au–Pt rounded UNCs.

## 4. Conclusions

In summary, we developed an efficient synthesis procedure for the production of Au–Pt HP-UNCs with undercoordinated surface atoms. The shape of the Au–Pt UNCs was largely dependent on the concentration of hydrazine used as reductant. The prepared Au–Pt HP-UNCs exhibited outstanding electrocatalytic performance toward MOR in comparison to their round counterparts and commercial Pt/C catalysts. The unique morphology and surface of the Au–Pt HP-UNCs can account for the enhanced electrocatalytic activity. These findings highlight that the precise control of the morphology and composition of metal NCs can lead to enhanced electrocatalytic performance in electrochemical oxidation reactions.
